# Trichurosis on a Conventional Swine Fattening Farm with Extensive Husbandry—A Case Report

**DOI:** 10.3390/pathogens11070775

**Published:** 2022-07-07

**Authors:** Moritz Bünger, René Renzhammer, Anja Joachim, Barbara Hinney, René Brunthaler, Mohamad Al Hossan, Julia Matt, Nora Nedorost, Christiane Weissenbacher-Lang, Lukas Schwarz

**Affiliations:** 1 University Clinic for Swine, Department for Farm Animals and Veterinary Public Health, University of Veterinary Medicine Vienna, 1210 Vienna, Austria; moritz.buenger@vetmeduni.ac.at (M.B.); 11829849@students.vetmeduni.ac.at (M.A.H.); lukas.schwarz@vetmeduni.ac.at (L.S.); 2 Institute of Parasitology, Department of Pathobiology, University of Veterinary Medicine Vienna, 1210 Vienna, Austria; anja.joachim@vetmeduni.ac.at (A.J.); barbara.hinney@vetmeduni.ac.at (B.H.); 3 Institute of Pathology, Department of Pathobiology, University of Veterinary Medicine Vienna, 1210 Vienna, Austria; rene.brunthaler@vetmeduni.ac.at (R.B.); julia.matt@vetmeduni.ac.at (J.M.); nora.nedorost@vetmeduni.ac.at (N.N.); christiane.weissenbacher-lang@vetmeduni.ac.at (C.W.-L.)

**Keywords:** *Trichuris suis*, pasture, extensive husbandry, diarrhoea, wasting, helminths, fattening pigs

## Abstract

Helminth infections of swine regain clinical and economic importance due to the increasing demand for pork from extensive husbandry. Infections with *Trichuris suis* in pigs can lead to wasting and diarrhoea. This was demonstrated by a case of clinical trichurosis on a conventional fattening farm, where pigs were kept on pasture. While all pre-fattening pigs, which had not been on the pasture yet, had a good body condition and firm faeces, diarrhoea and poor body condition were observed in approximately half of the fattening pigs kept on pasture. Rectally collected faecal samples from all animals were investigated using faecal flotation. High numbers of *T. suis* eggs were detected in 17 out of 32 faecal samples, while all samples from pre-fattening pigs were negative. The highest number of eggs per gram of faeces was 778,000. Two out of three environmental samples were also positive for *T. suis* in faecal flotation. This case demonstrates that *T. suis* must be considered as an enteropathogen in pigs kept on pasture, as favourable environmental conditions, and the lack of removal of faeces from a pasture can lead to the accumulation of large numbers of infective eggs in the pigs’ surroundings.

## 1. Introduction

*Trichuris suis*, the whipworm of swine, has a worldwide distribution and is able to infect pigs as well as numerous primate species, including humans [1]. Eggs of *Trichuris* can be identified easily by their lemon-shaped appearance and two characteristic bipolar plugs. Infected pigs shed *T. suis* eggs approximately five to eight weeks after infection [2]. Infective first-stage larvae develop within at least three weeks and, depending on temperature, humidity and soil type, can remain infective in the environment for up to 15 years [3]. Transmission of *T. suis* is most frequently a result of ingestion of soil contaminated with infective eggs [3]. After the ingestion of such eggs, the egg plugs are digested, and the larvae hatch in the ileum, caecum and colon, where they invade the mucosal tissue [3]. In the course of four moults, larvae reach their adult stage within six to eight weeks after infection [4]. Adult stages are characterised by a thinner anterior part, which accounts for approximately two-thirds of the total body length and is stitched into the intestinal mucosa of the caecum and proximal colon, and a thicker posterior part protruding into the intestinal lumen [3]. Inconsistent egg production reaches its peak approximately seven weeks post-infection and decreases rapidly within the consecutive five weeks, probably due to a developing immune response [2]. High infection doses of *T. suis* can lead to diarrhoea, anorexia, anaemia and reduced average daily weight gain [3]. Additionally, blood or mucus might be present in the faeces of animals prior to death [3]. Gross lesions due to *T. suis* infections include the presence of adult whipworms, watery to mucoid intestinal contents, typhlitis and colitis characterised by ulceration, oedema and haemorrhages [5]. Faecal flotation is still widely applied for confirmation of infection. However, false-negative results may occur due to inconsistent shedding and long prepatent periods [5]. Furthermore, quantitative methods such as the McMaster method provide the possibility to count the number of eggs per gram of faeces (EPG), which can be used to estimate the level of environmental contamination [6]. Application of anthelmintic drugs such as fenbendazole, flubendazole or febantel for at least five to fifteen days provides sufficient efficacy against *Trichuris* in most cases [7,8] but has no effect on infective eggs present in the environment, and reinfections will occur regularly.

## 2. Case Presentation

### 2.1. Description of the Farm

The case farm was a two-site conventional finishing farm with a capacity of approximately 60 fattening pigs (Duroc) kept on a pasture in Lower Austria. Weaners with approximately 30 kg of body weight were regularly purchased from a single piglet producer, where they had been vaccinated against Porcine Circovirus 2, *Mycoplasma hyopneumoniae* and *Lawsonia intracellularis*. All purchased pigs were kept in a semi-open stable on concrete floor with deep straw bedding in groups of approximately 20 animals before they were integrated into the herd on pasture six weeks later. The farm used two equivalent areas of pasture in a yearly rotation (in total 1.70 ha). Additionally, there was also a closed stable with deep straw bedding, where fattening pigs reaching the corresponding body weight for slaughter were transferred to from pasture. Neither vaccinations nor any routine antibiotic or anthelmintic treatments had been performed on the farm. While growth rates of the pigs had initially decreased slowly, during the summer of 2021, the farmer observed an increase in severe wasting and anorectic pigs with diarrhoea, followed by increased mortality.

### 2.2. Section and Diagnostic Workup of Four Fattening Pigs

**Figure 1:**
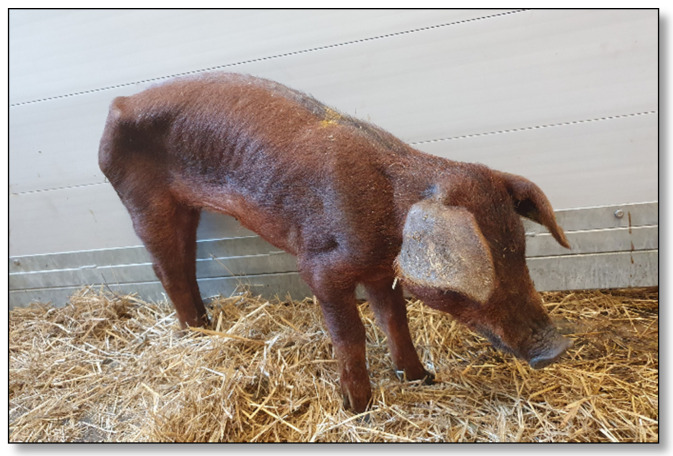
One of four fattening pigs with a poor body condition sent for diagnostic workup at the University Clinic for Swine.

**Figure 2:**
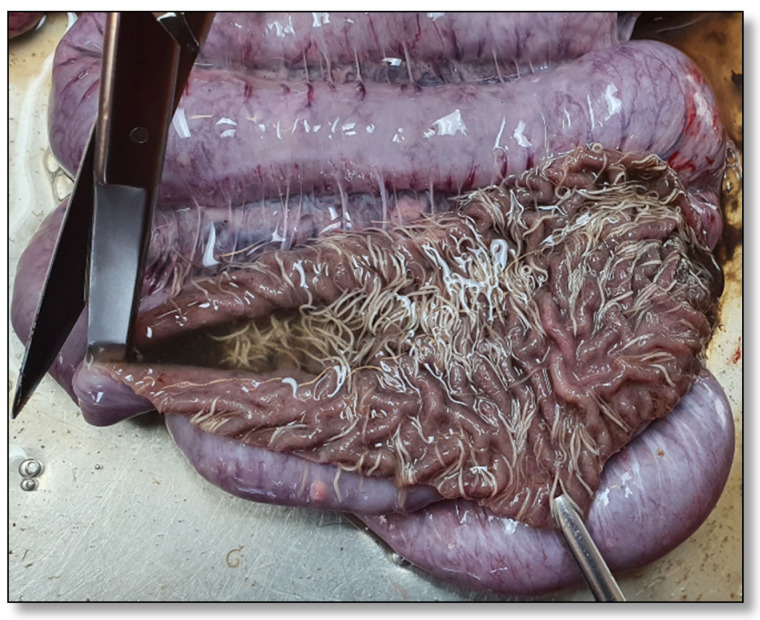
Large numbers of adult *Trichuris suis* attached to the intestinal mucosa of the colon of one of the necropsied pigs.

In August 2021, four fattening pigs (19.5, 23, 23.5 and 30 kg) were sent to the University Clinic for Swine, Vetmeduni Vienna, Austria, for diagnostic workup. All four animals had poor body conditions (Figure 1). Rectal temperatures of the animals were within the physiological range, except for one pig which suffered from hypothermia (35.3 °C). Two pigs also suffered from kyphosis, anaemia and dyspnoea. All four animals were euthanised for necropsy according to appropriate welfare procedures. During necropsy, high numbers of adult whipworms attached to the mucosa of the ileum, caecum and particularly the colon were observed in all animals (Figure 2).

**Figure 3:**
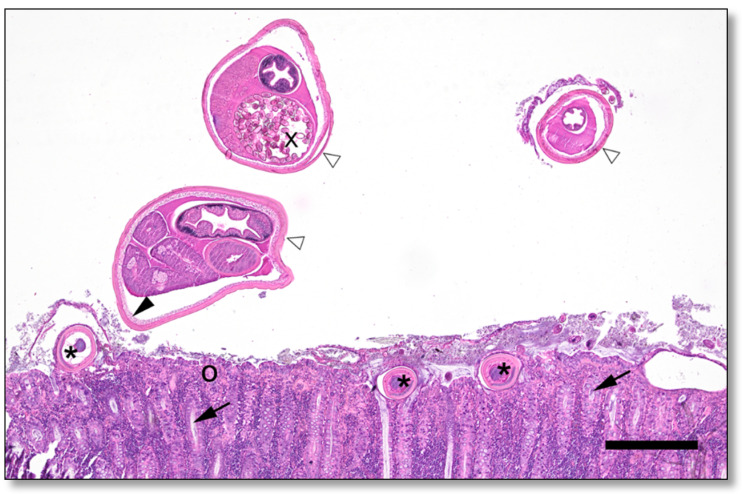
Histologic section (haematoxylin and eosin staining; bar = 400 µm) of colonic mucosa from one of the necropsied pigs. Asterisk: anterior end of a worm embedded in the intestinal epithelium, black arrowhead: bacillary band, white arrowheads: posterior end of a worm in the intestinal lumen, x: eggs, black arrow: crypts of Lieberkühn, o: Lamina propria mucosae.

In histopathologic examination, moderate infiltration with eosinophilic granulocytes in the lamina propria and hyperplasia of goblet cells were observed in all parts of the intestine affected by whipworms. The cranial parts of the whipworms embedded in the mucosa were detectable in the histopathologic investigation in multiple locations (Figure 3).

Additionally, hyperplasia of the bronchoalveolar lymphatic tissue was observed in the lungs of two animals. PCRs for detection of *L. intracellularis*, *Brachyspira hyodysenteriae* and *B. pilosicoli* were negative in scrapings from the ileal and colonic mucosa (Table 1, Supplementary File S1). *Salmonella* spp. were not detectable via enrichment with tetrathionate and consecutive microbiological examination of the tonsil, caecum and ileocaecal lymph node. While *M*. *hyopneumoniae* DNA was detectable in pneumonic lung tissue of two animals, PCR for the detection of Porcine Reproductive and Respiratory Syndrome Virus (PRRSV) RNA was negative in the lungs and lymphatic tissue of all animals. Upon faecal flotation with zinc sulphate, a high number of *T*. *suis* eggs was observed in faecal samples from all four animals. For details on the methods, refer to Table 1.

### 2.3. Farm Visit, Clinical Examination and Sampling

After the confirmation of trichurosis as the cause of colitis and poor body condition in the necropsied animals, a farm visit was conducted in order to evaluate the worm burden of the animals on the farm in more detail and to evaluate possible therapeutic and prophylactic measures for the farm. The body condition of 34 fattening pigs on pasture and 20 pre-fattening pigs in the semi-open pre-fattening stable was assessed (Table 2). Faeces (~10 g/animal) were sampled directly from the rectum, and faecal consistency was grossly evaluated for all animals. Faecal samples could be collected in sufficient amounts from all but two pigs, resulting in a total number of 52 faecal samples. Additionally, soil samples from the wallow, and the feeding area and a straw sample from the finisher unit were taken as well.

Faecal flotation for detection of parasitic stages (including determination of EPG in samples with a high grade of eggs upon flotation) and PCRs for the detection of *L. intracellularis*, *B. hyodysenteriae* and *B. pilosicoli* DNA were performed from 52 faecal samples and environmental samples, as described in Supplementary File S1.

### 2.4. Results

While all pre-fattening pigs had a good (n = 15) or moderate (n = 5) body condition, more than one-third of the fattening pigs kept on pasture had either a poor or bad body condition (Table 2). Out of all 20 pre-fattening pigs, the faecal consistency of 18 pigs was firm, while pasty faeces were recorded for the other two pigs (Table 2). Altogether, six fattening pigs had semiliquid diarrhoea, and eight animals had liquid diarrhoea (Table 2).

Adult whipworms were visible in faecal samples from five fattening pigs. While no *T. suis* eggs or stages of other helminth species were detected in the 20 faecal samples from the pre-fattening pigs, all but three samples of the 32 faecal samples from the fattening pigs kept on pasture were positive for *Trichuris* at flotation, 17 of them in a “high” grade (>10 eggs per coverslip). High numbers of *T. suis* eggs were detectable in faecal samples from twelve out of 14 fattening pigs with liquid or semiliquid diarrhoea. Nine out of twelve animals with a bad or poor body condition also had high numbers of detectable *T. suis* eggs in their faecal samples.

The median EPG determined in the 17 samples being highly positive for *Trichuris* was 23,750 (maximum: 778,000; Table 3). Of those 17 pigs, five had pasty faeces, five had semiliquid and seven had liquid diarrhoea. Firm faecal consistency was not observed in this group (Table 3).

Low numbers of oocysts from *Eimeria* spp. were detected in one faecal sample (ID 1; *T. suis*: 1,150 EPG). Another sample which was negative for *T. suis* contained a large amount (>10 cysts/coverslip) of *Balantidium coli* cysts. While DNA of neither *B. hyodysenteriae* nor *B. pilosicoli* was detectable by PCR, *L. intracellularis* DNA was detected in faecal samples from two animals which both had poor body conditions and diarrhoea.

Flotation of the straw sample from the finisher unit and the soil sample from the feeding area also revealed high numbers of *T. suis* eggs, whereas no eggs were detectable in the soil sample from the wallow.

All animals on pasture were immediately treated with a commercial formulation of fenbendazole for pigs at the recommended dose of 5 mg/kg body weight for seven days. Subsequently, the farmer reported a marked improvement in body conditions and an absence of further losses. The treatment was repeated after five weeks to further decrease the worm burden in the herd. Since eggs of *T. suis* were not detectable in faeces from pre-fattening piglets, no measures were taken for this age group. Regular treatment of all pigs every five weeks to prevent trichurosis was recommended.

## 3. Discussion

This case report demonstrates that infections with *T. suis* can cause severe clinical symptoms and economic losses if proper control measures, including hygiene and anthelmintic treatment, are neglected, especially on farms with free-range husbandry on pasture. Therefore, *T. suis* infections will probably regain importance in pig production due to the increasing demand for pork derived from extensive and free-range husbandry. Despite the fact that this branch of pig production is associated with a high standard of animal welfare, animal health and its impact on animal welfare are discussed far less frequently and sometimes even stand in contrast to common expectations of consumers due to the bad reputation of routine antibiotic therapy [15,16]. This case highlights the importance of animal health care (including proper anthelmintic treatment after correct diagnosis) for animal welfare as well as sustainable productivity.

On the presented farm, approximately 35% of the observed fattening pigs on pasture had a poor or bad body condition, whereas the body condition of the pre-fattening pigs was predominantly good. Since wasting and diarrhoea only occurred after several weeks on pasture, and anthelmintic treatment quickly relieved the pigs’ condition, *T. suis* was very likely the primary cause of the observed clinical symptoms on this farm. High excretion rates of *T. suis* eggs in three fattening pigs with good body conditions were presumably due to a recent infection, compared to pigs with poorer body conditions. The presence of diarrhoea in 24 out of 34 examined fattening pigs also emphasises that infections with *T. suis* should be included as a common differential diagnosis in cases of diarrhoea in pigs, especially in extensive husbandry systems.

Since eggs of *T. suis* were not detected in faeces from the pre-fattening pigs, which had been purchased five weeks prior to sampling, first contact with *T. suis* supposedly happened after animals had been moved on the pasture. This is not surprising since husbandry on pasture significantly increases the risk of infections with various helminths [17].

The detection of *T. suis* eggs in environmental samples further confirmed high contamination of the pasture, as previously described for *T. suis*-affected farms [18]. Interestingly, the soil sample from the wallow was negative in flotation. This might be due to a patchy distribution of eggs in this part of the pasture due to soil movements by the wallowing pigs.

Due to the high numbers and long persistence of *T. suis* eggs in the soil, decontamination of the pasture is not feasible [3] and was, therefore, not part of the recommended control strategy for this farm. In addition, yearly pasture rotation between two areas is probably not sufficient to interrupt transmission. To ensure proper decontamination over time, the areas would have to stay unused for several years, which is neither feasible nor economically sensible for most outdoor farming systems. When planning for extensive pig husbandry on pasture, sufficient space for multiple alternating pastures is highly recommended to decrease parasite density on pasture and to prevent a high worm burden. Another strategy could be to start outdoor farming with pigs which are either negative for helminths or are treated prior to husbandry on pasture. This would imply regular and repeated copromicroscopical examinations of pig faeces for the presence of parasitic stages in every batch. However, this is no guarantee for negativity, since several helminthic eggs are shed irregularly or (during prepatency) not at all. This underlines the impact of helminths on outdoor farming of fattening pigs, as it necessitates the development of a proper strategy for controlling parasites not only during but also prior to starting outdoor husbandry on pasture.

Although several case reports of clinical trichurosis in pigs provide data on the number of excreted EPG of infected pigs [3,8,19,20,21], an excretion rate of 778,000 EPG has not been reported so far. It was once estimated that one female *T. suis* is able to produce approximately 6500 to 7800 eggs per day, which would result in 6–8 EPG produced by each female [22]. Thus, we carefully assume that the fattening pig with 778,000 EPG was affected with over 100,000 female *T. suis*.

An increase in eosinophilic granulocytes in the large intestine is compatible with previous findings of experimental infections with *T. suis* [23]. Altogether, all histologic findings including multiple sections of adult whipworms further underpin the diagnosis of clinical trichurosis.

Since neither DNA of *B. hyodysenteriae* nor *B. pilosicoli* could be detected via PCR, and *Salmonella* spp. was not isolated via microbiological examination, some of the most common and relevant causative agents of diarrhoea during the fattening period could be ruled out [24]. While *L. intracellularis* DNA was detected in two animals with diarrhoea, there was no evidence for clinical manifestations, especially because *L. intracellularis* is ubiquitous in most European swine stocks [25]. Furthermore, since DNA was only detected in two out of 34 investigated samples, *L. intracellularis* could be neglected as a primary cause of clinical disease in this case herd. Nevertheless, accelerating effects between *T. suis* and *L. intracellularis* could not be ruled out, but they were evaluated as neglectable for this case herd since in case of cumulative pathogenicity of *T. suis* and *L. intracellularis*, higher in-herd prevalence rates may be expected and diagnosed. *Balantidium coli* was detected in the faecal sample of one pig. However, the impact of *Balantidium coli* on pigs has not been completely elucidated yet. While several authors assume that it is a non-pathogenic coloniser of porcine intestines, there is evidence that colonisation with *Balantidium coli* can lead to mild colitis [26,27,28].

## 4. Conclusions

Altogether, this case confirms that heavy *T. suis* infections can lead to severe clinical symptoms in pigs, even though trichurosis has become rarer with the introduction of closed stables and slatted floors. Therefore, farmers and veterinarians should not abandon regular anthelmintic treatment and routine parasitological examination, particularly on farms with extensive husbandry on pasture, to ensure animal health, animal welfare and farm productivity.

## Figures and Tables

**Table 1:** Laboratory diagnostic methods for pathogen detection applied in this study.

Pathogen	Detection Method	Specimen	Reference
*Lawsonia intracellularis, Brachyspira hyodysenteriae, Brachyspira pilosicoli*	PCR	Ileal and Colonic Mucosal Scrapings	Supplementary File S1 [9,10]
*Salmonella* spp.	Microbiological examination	Tonsil, Caecum, Ileocaecal Lymph Node	[11]
*Mycoplasma hyopneumoniae*	PCR	Lung	[12]
Porcine Reproductive and Respiratory Syndrome Virus	qRT—PCR	Lung, Tonsil, Tracheobronchial Lymph Node	[13]
Helminth eggs	Flotation (saturated ZnSO_4_); modified McMaster technique	Faeces	[14]

**Table 2:** Number of pigs with different body conditions and faecal consistency.

Body Condition	Fattening Pigs (n = 34)	Pre-Fattening Pigs (n = 20)
Bad	7 (21%)	0 (0%)
Poor	5 (15%)	0 (0%)
Moderate	13 (38%)	5 (25%)
Good	9 (26%)	15 (75%)
**Faecal Score**	**Fattening Pigs (n = 34)**	**Pre-Fattening Pigs (n = 20)**
Liquid	8 (24%)	0 (0%)
Semiliquid	6 (18%)	0 (0%)
Pasty	10 (29%)	2 (10%)
Normal	10 (29%)	18 (90%)

**Table 3:** EPG faeces of 17 fattening pigs with a high number of *T. suis* eggs in flotation and the respective faecal consistency and body condition scores.

ID	Faecal Consistency	Body Condition	*Trichuris* EPG (McMaster)	ID	Faecal Consistency	Body Condition	*Trichuris* EPG (McMaster)
1	Liquid	Bad	1150	10	Liquid	Good	26,700
2	Semiliquid	Bad	3800	11	Pasty	Good	32,560
3	Pasty	Bad	6200	12	Liquid	Poor	33,680
4	Semiliquid	Poor	11,500	13	Semiliquid	Bad	35,450
5	Semiliquid	Good	13,250	14	Pasty	Moderate	35,800
6	Liquid	Moderate	14,250	15	Pasty	Moderate	44,500
7	Liquid	Moderate	20,550	16	Liquid	Moderate	98,870
8	Liquid	Bad	22,450	17	Semiliquid	Bad	778,000
9	Pasty	Poor	23,750				

## Data Availability

Not applicable.

## Supplementary Materials

The following are available online at https://www.mdpi.com/article/10.3390/pathogens11070775/s1, File S1: Methods of PCR for detection.
