# Supplementary material for: Trichurosis on a Conventional Swine Fattening Farm with Extensive Husbandry—A Case Report
Source: Pathogens. 2022 Jul 7;11(7):775. doi: 10.3390/pathogens11070775 (PMC9315933; doi:10.3390/pathogens11070775)
Supplement: Supplementary file 1 [file pathogens-11-00775-s001.zip › pathogens-1796719-supplementary.pdf]

Supplementary File S1

Methods

DNA extraction

Prior to PCR DNA was extracted from scrapings from ileal and colonic mucosa using the Nexttec™ 1-Step DNA Isolation Kit for Tissue and Cells (Nexttec™ Biotechnologie GmBH, Hilgertshausen, Germany) and from 200 mg faeces or faecal swabs using the QIAamp® Fast DNA Stool Mini Kit (Qiagen, Vienna, Austria) according to the manufacturers' instructions. DNA was eluted in 100 (Nexttec™ 1-Step DNA Isolation Kit for Tissue and Cells) or 200 µl (QIAamp® Fast DNA Stool Mini Kit) buffer.

Duplex PCR for the detection of Brachyspira hyodysenteriae and Brachyspira pilosicoli

For the detection of *Brachyspira* (*B.*) *hyodysenteriae* and *B. pilosicoli* the protocol of La et al. (2003) was modified. The PCR reaction master mixture consisted of 12.5 µL Qiagen Multiplex PCR Kit (Qiagen, Vienna, Austria), 0.5 µl (10 pM) of each primer (*B. hyodysenteriae*: H1: 5′-ACT AAA GAT CCT GAT GTA TTT G-3′; H2: 5′-CTA ATA AAC GTC TGC TGC-3′; *B. pilosicoli*: P1: 5′-AGA GGA AAG TTT TTT CGC TTC-3′, P2: 5′-GCA CCT ATG TTA AAC GTC CTT G-3′), 1 µL template DNA extracted from mucosal scrapings or 2 µl template DNA extracted from faecal swabs and distilled water to a total volume of 25 µL per reaction. The cycler program started with an initial heat denaturation step at 95 °C for 15 min, followed by 35 cycles at 94 °C for 30 s, 58 °C for 90 s, and 72 °C for 90 s, and a final extension step at 72 °C for 10 min.

PCR for the detection of Lawsonia intracellularis

For the detection of *Lawsonia* (*L.*) *intracellularis* the outer primers of the nested PCR of Jones et al. (1993) were used. The PCR reaction master mixture consisted of 12.5 µL Kapa 2G Fast HotStart ReadyMix (Sigma-Aldrich Handels Gmbh, Vienna, Austria), 1 µl (10 pM) of each primer (Law A (F): 5′-TAT GGC TGT CAA ACA CTC CG-3′, Law B (R): 5′-TGA AGG TAT TGG TAT TCT CC-3′), 1 µL template DNA extracted from mucosal scrapings or 2 µl template DNA extracted from faecal swabs, 1 µl $MgCl_2$ (Peqlab, VWR, Vienna, Austria) and distilled water to a total volume of 25 µL per reaction. The cycler program started with an initial heat denaturation step at 95 °C for 3 min, followed by 40 cycles at 95 °C for 15 s, 55 °C for 15 s and 72 °C for 25 s, and a final extension step at 72 °C for 10 min.

Gel electrophoresis

The successful amplification was checked by gel electrophoresis by analyzing an aliquot of 10 µL of each PCR product on a 2% Tris acetate-EDTA-agarose gel. The agarose gel was stained (ROTI® GelStain; Lactan, Graz, Austria), and bands were detected (Molecular Imager, GEL DOC™ XR+, BioRad Laboratories, Vienna, Austria). PCR products of the expected sizes (*B. hyodysenteriae*: 354 bp, *B. pilosicoli*: 823 bp, *L. intracellularis*: 319 bp) were evaluated positive.

References

La, T., Phillips, N.D., Hampson, D.J. Development of a Duplex PCR Assay for Detection of *Brachyspira hyodysenteriae* and *Brachyspira pilosicoli* in Pig Feces. *Journal of Clinical Microbiology* **2003**, 41(7), 3372–3375, doi:10.1128/JCM.41.7.3372–3375.2003.

Jones, G.F., Ward, G.E., Murtaugh, M.P., Lin, G., Gebhart, C.J. Enhanced Detection of Intracellular Organism of Swine Proliferative Enteritis, Ileal Symbiont Intracellularis, in Feces by Polymerase Chain Reaction. *Journal of Clinical Microbiology* **1993**, 31(10), 2611–2615, doi: 10.1128/jcm.31.10.2611-2615.1993.
